# Asymmetric segregation of template DNA strands in basal-like human breast cancer cell lines

**DOI:** 10.1186/1476-4598-12-139

**Published:** 2013-11-15

**Authors:** Wenyu Liu, Gajan Jeganathan, Sohrab Amiri, Katherine M Morgan, Bríd M Ryan, Sharon R Pine

**Affiliations:** 1 Rutgers Cancer Institute of New Jersey, Rutgers, The State University of New Jersey, 195 Little Albany Street, New Brunswick, New Jersey, USA; 2 Laboratory of Human Carcinogenesis, Center for Cancer Research, National Cancer Institute, Bethesda, Maryland, USA; 3 Robert Wood Johnson Medical School, Rutgers, The State University of New Jersey, New Brunswick, New Jersey, USA

**Keywords:** Non-random chromosome segregation, Asymmetric cell division, Immortal DNA strand hypothesis, Breast cancer, Cancer stem cells

## Abstract

**Background and methods:**

Stem or progenitor cells from healthy tissues have the capacity to co-segregate their template DNA strands during mitosis. Here, we set out to test whether breast cancer cell lines also possess the ability to asymmetrically segregate their template DNA strands via non-random chromosome co-segregation, and whether this ability correlates with certain properties attributed to breast cancer stem cells (CSCs). We quantified the frequency of asymmetric segregation of template DNA strands in 12 human breast cancer cell lines, and correlated the frequency to molecular subtype, CD44^+^/CD24^-/lo^ phenotype, and invasion/migration ability. We tested if co-culture with human mesenchymal stem cells, which are known to increase self-renewal, can alter the frequency of asymmetric segregation of template DNA in breast cancer.

**Results:**

We found a positive correlation between asymmetric segregation of template DNA and the breast cancer basal-like and claudin-low subtypes. There was an inverse correlation between asymmetric segregation of template DNA and Her2 expression. Breast cancer samples with evidence of asymmetric segregation of template DNA had significantly increased invasion and borderline significantly increased migration abilities. Samples with high CD44^+^/CD24^-/lo^ surface expression were more likely to harbor a consistent population of cells that asymmetrically segregated its template DNA; however, symmetric self-renewal was enriched in the CD44^+^/CD24^-/lo^ population. Co-culturing breast cancer cells with human mesenchymal stem cells expanded the breast CSC pool and decreased the frequency of asymmetric segregation of template DNA.

**Conclusions:**

Breast cancer cells within the basal-like subtype can asymmetrically segregate their template DNA strands through non-random chromosome segregation. The frequency of asymmetric segregation of template DNA can be modulated by external factors that influence expansion or self-renewal of CSC populations. Future studies to uncover the underlying mechanisms driving asymmetric segregation of template DNA and dictating cell fate at the time of cell division may explain how CSCs are maintained in tumors.

## Background

In mature organisms, tissue homeostasis is maintained through a dynamic balance between symmetric and asymmetric cell divisions of its stem or progenitor cells. Asymmetric cell division produces unequal daughter cells; one retains its stem cell properties and the other acquires a more differentiated phenotype. Similar to normal stem cells, cancer stem cells (CSCs) are also able to asymmetrically self-renew. However, CSC divisions favor excessive symmetric self-renewal that drives expansion of the CSC pool [1-4]. Understanding the mechanisms driving cell fate decisions during cell division of CSCs could lead to therapies designed to deplete the CSC pool.

Non-random co-segregation of sister chromatids during mitosis, also known as asymmetric segregation of template DNA strands, has been reported in various tissues, including mouse embryonic fibroblasts [5], hair follicles [6], nasopharyngeal epithelium [7], intestinal epithelium [8], mammary gland epithelium [9,10], and skeletal muscle [11,12]. Several hypotheses have arisen to explain these observations including reduction of cancer-promoting DNA replication errors, and epigenetic modification of daughter cells [13], although the functional relevance has not yet been determined experimentally. Not all tissues harbor cells that asymmetrically segregate their template DNA [14,15], suggesting that asymmetric DNA segregation may be restricted to certain cell lineages. We previously reported that asymmetric segregation of template DNA is retained in human non-small cell lung cancer (NSCLC) cell lines and short-term cultures of primary lung tumors. We showed that asymmetric segregation of template DNA in NSCLC results in divergent fates of the daughter cells, are enriched within the CD133+ CSC fraction, and can be modulated by changes in the micro-environment [16]. Subsequently, asymmetric segregation of template DNA was reported in liver, gastric and colon cancer cell lines [4,17-19], as well as cultures of malignant peritoneal mesothelioma stem cells [20]. Furthermore, label-retaining cells were detected in nasopharyngeal carcinoma tissues [7]. Here, we set out to determine if asymmetric segregation of template DNA occurs in human breast cancer cell lines, if the frequency of asymmetric segregation of template DNA correlates with certain CSC-like features, and if it can be modulated by external factors that influence expansion or self-renewal of CSC populations.

## Results

### Breast cancer cells asymmetrically segregate their template DNA strands during cell division

**Figure 1:**
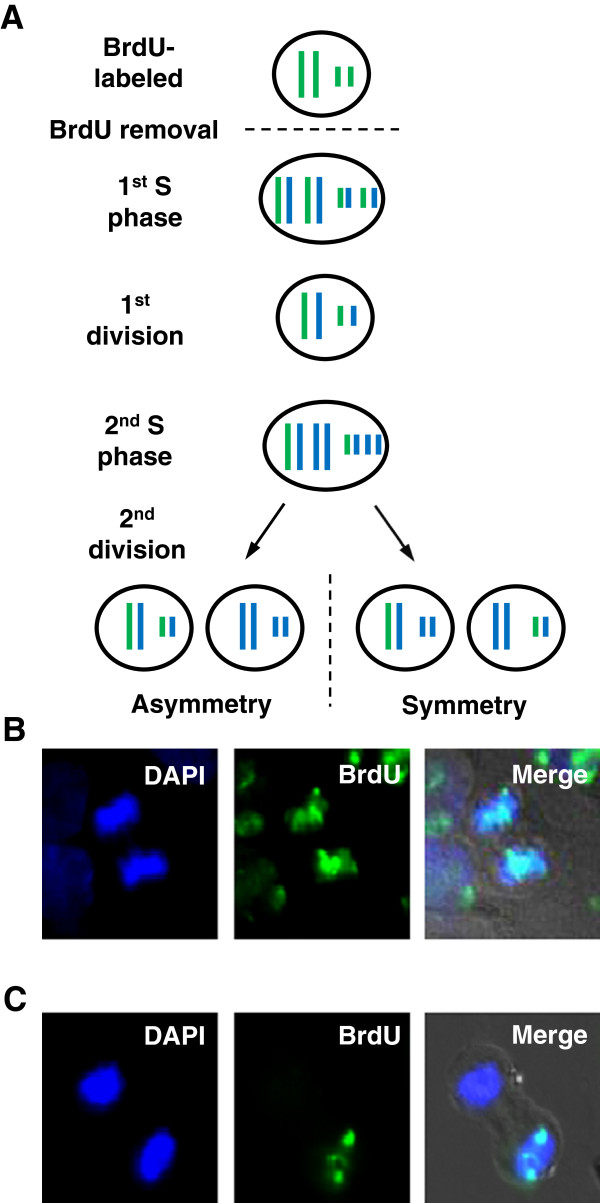
**Human breast cancer cell lines asymmetrically segregate their template DNA strands. (A)** Schematic representation of the BrdU labeling assay. Chromosomes were labeled with BrdU (shown as green lines). BrdU was then removed from the media and cells were cultured for two cell divisions. Newly synthesized DNA is shown as blue lines. Due to semi-conservative DNA replication, asymmetric segregation of template DNA is apparent at the 2nd anaphase. **(B)** Representative images of a HCC1143 cell in anaphase with random segregation of its template DNA to both sets of chromosomes. **(C)** Representative images of an anaphase HCC1143 cell with template DNA segregated exclusively to one set of chromosomes, on the right bottom. Blue: DAPI-labeled DNA. Green: BrdU-labeled DNA.

**Figure 2:**
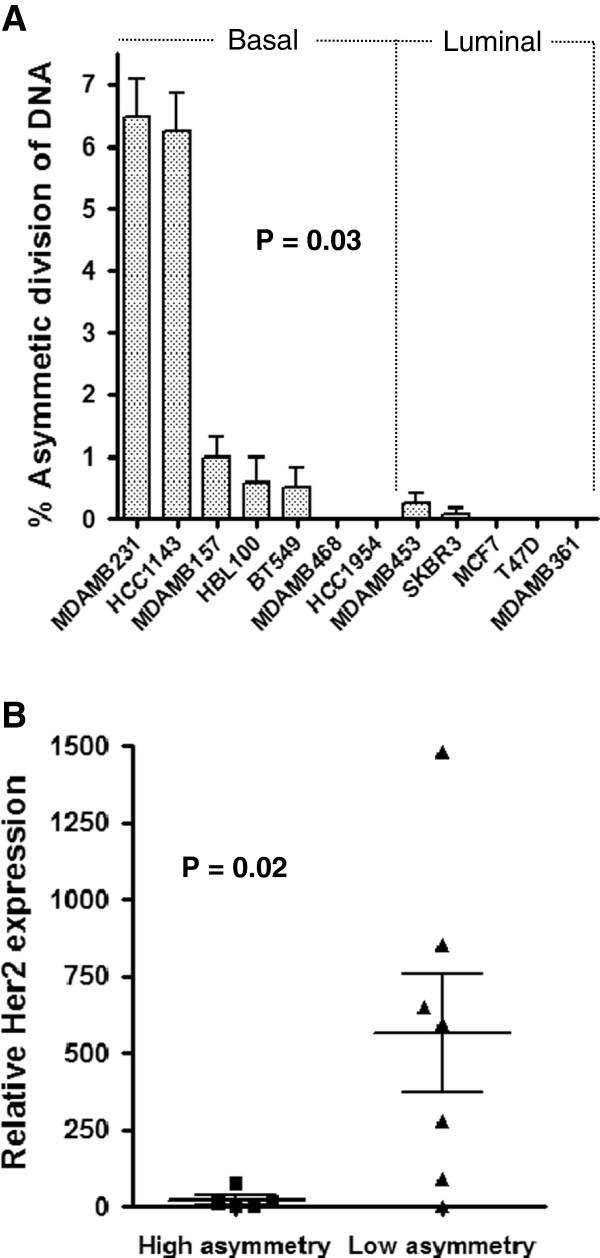
**Frequency of asymmetric segregation of template DNA and correlation with Her2 expression. (A)** Percentages of cells with asymmetric division of BrdU-labeled DNA templates are shown for 12 breast cancer cell lines. Analyses were performed in triplicates in triplicates and at least two independent experiments were performed. Basal cell lines had a higher frequency of ACD as compared to luminal cell lines (P = 0.03, Fisher’s exact test) **(B)** The breast cancer cell lines were divided into two groups according to the percentage of BrdU asymmetric division observed, high asymmetry (mean, ≥ 0.5%, which was the limit of quantifiable detection) and low asymmetry (mean, <0.5%). Relative Her2 expression, as assessed by flow cytometry in at least two independent experiments, is shown as mean fluorescence intensity. Cell lines with high asymmetry had lower Her2 expression as compared to cell lines with low asymmetry (P = 0.02, Mann–Whitney test). Error bars are standard error of the mean.

To verify whether human breast cancer cells asymmetrically segregate their template DNA strands, classical Bromodeoxyuridine (BrdU) pulse-labeling strategies were performed as described [16] (Figure 1A). Twelve human breast cancer cell lines were cultured in the presence of BrdU for at least 2 weeks to ensure that all the cells and DNA strands were labeled. Staining cells with anti-BrdU antibody at the second anaphase following BrdU removal revealed that the majority of cells segregated their DNA templates randomly (Figure 1B). As was previously observed in NSCLC [16], a low percentage of anaphase cells in several cell lines showed evidence of BrdU-labeled template chromosomes that were segregated exclusively to one side of the metaphase plate, whereas the other set of chromosomes was completely devoid of BrdU label (Figure 1C). However, in contrast to NSCLC in which the vast majority of samples showed evidence for asymmetric segregation of template DNA [16], we observed quantifiable asymmetric segregation of template DNA in only 5 of the 12 breast cancer cell lines (Figure 1A). The frequency of non-random chromosome segregation ranged from 6.47 ± 0.62% to 0.50 ± 0.32% (Figure 2A, Table 1). We did not observe any cells with asymmetric segregation of template DNA among 1,200 anaphases each in MDA-MB-468, HCC1954, MCF7, T47D, or MDA-MB-361 cells. Two additional cell lines, MDA-MB-453 and SKBR3, had sporadic low-level asymmetric segregation of template DNA that were observed only once or twice among several independent experiments, suggesting that the actual frequency was just at or below the detection threshold and could not be accurately quantified. In all subsequent analyses, cell lines were grouped into those with quantifiable levels of asymmetric segregation of template DNA versus those with very low or no detectable levels.

**Table 1:** Frequencies of BrdU asymmetric division, surface marker expression and functional assays in breast cancer cell lines

**Cell line**	**% Asymmetric**	**% CD44**^**+**^**/**	**HER2**	**No. invaded**	**No. migrated**	**Soft agar**
	cell division*	**CD24**^**-/lo**^	**(MFI)**	cells*	cells*	colony no.*
**Basal**						
MDAMB231	6.5 ± 0.6	99.9	9.4	83.5 ± 0.0	75.0 ± 5.9	45 ± 7
HCC1143	6.3 ± 0.6	16.4	0	0.9 ± 0.3	4.9 ± 0.5	0
MDAMB157	1.0 ± 0.3	97.4	73.5	32.0 ± 1.7	50.4 ± 2.6	0
HBL100	0.6 ± 0.4	81.3	16.4	14.7 ± 1.5	9.3 ± 1.1	99 ± 16
BT549	0.5 ± 0.3	90.3	0.4	14.1 ± 3.7	171.0 ± 8.2	0
MDAMB468	0	1.6	2.9	3.2 ± 0.3	42.6 ± 1.4	390 ± 35
HCC1954	0	2.5	851.9	20.4 ± 2.9	46.8 ± 2.5	1 ± 1
**Luminal**						
MDAMB453	≤ 0.25	0	648.9	1.9 ± 0.2	5.9 ± 0.7	0
SKBR3	≤ 0.25	0	1478.8	0.4 ± 0.1	3.7 ± 0.4	0
MCF7	0	3.8	279.7	0.2 ± 0.1	2.8 ± 0.6	1 ± 0
T47D	0	0	89.7	0.6 ± 0.2	6.4 ± 0.3	13 ± 4
MDAMB361	0	0.8	586.2	0.3 ± 0.1	3.3 ± 0.4	0

*Values were expressed as mean ± standard error of the mean.

*MFI* Mean fluorescence intensity.

### Asymmetric segregation of template DNA varies by molecular breast cancer subtype

Breast cancer is a molecularly and phenotypically diverse disease composed of distinct biological subtypes with differing responses to therapy. To assess if the wide variations in frequency of asymmetric segregation of template DNA could be due to differences in breast cancer subtypes, we grouped the 12 cell lines into basal-like and luminal [21]. The cell lines with consistently observed asymmetric segregation of template DNA were basal-like, whereas none of the luminal breast cancer cell lines had consistent asymmetric segregation of template DNA (P = 0.03) (Figure 2A). The basal-like phenotype can be further classified into a claudin-low molecular subtype [22] that is enriched for markers associated with breast CSCs [23]. Four of the cell lines in our panel, MDA-MB-231, MDA-MB-157, HBL100, and BT549 were previously classified as claudin-low [23]. The claudin-low cell lines in our panel were significantly more likely to harbor a consistent population of cells with asymmetric segregation of template DNA as compared to the remainder of the basal-like and luminal cell lines (P = 0.01). This suggests that asymmetric chromosome segregation in breast cancer is molecular subtype-dependent and may be enriched in claudin-low breast tumors.

We next considered specific features that correspond with breast cancer subtypes that may participate in regulating self-renewal. Her2 is overexpressed more frequently in the luminal subtype, and is associated with poor prognosis and increased breast cancer recurrence [24]. Furthermore, in the ErbB2 transgenic mouse model of breast cancer, Her2 is constitutively activated in the mammary epithelium, resulting in expansion of mammary CSCs through decreased asymmetric and increased symmetric self-renewing divisions [1]. Consistent with the ErbB2 mouse model of breast cancer, we observed a positive correlation between low Her2 mean fluorescence intensity (MFI) levels and breast cancer cell lines with quantifiable asymmetric segregation of template DNA (P = 0.02) (Figure 2B and Table 1).

The p53 pathway is known to participate in increased asymmetric self-renewal. Wild-type p53 expression was reported to increase non-random chromosome segregation in fibroblasts and epithelial cells [25], and there was an expansion of primary premalignant mouse mammary stem cells when TP53 was deleted [1]. Only two of the cell lines in our breast cancer panel (HBL100 and MCF7) were wild-type for TP53 [26] ( http://p53.free.fr/), and therefore we did not assess the correlation between p53 status and asymmetric segregation of template DNA in this study.

### Correlation of asymmetric segregation of template DNA frequency with CSC phenotype and function

**Figure 3:**
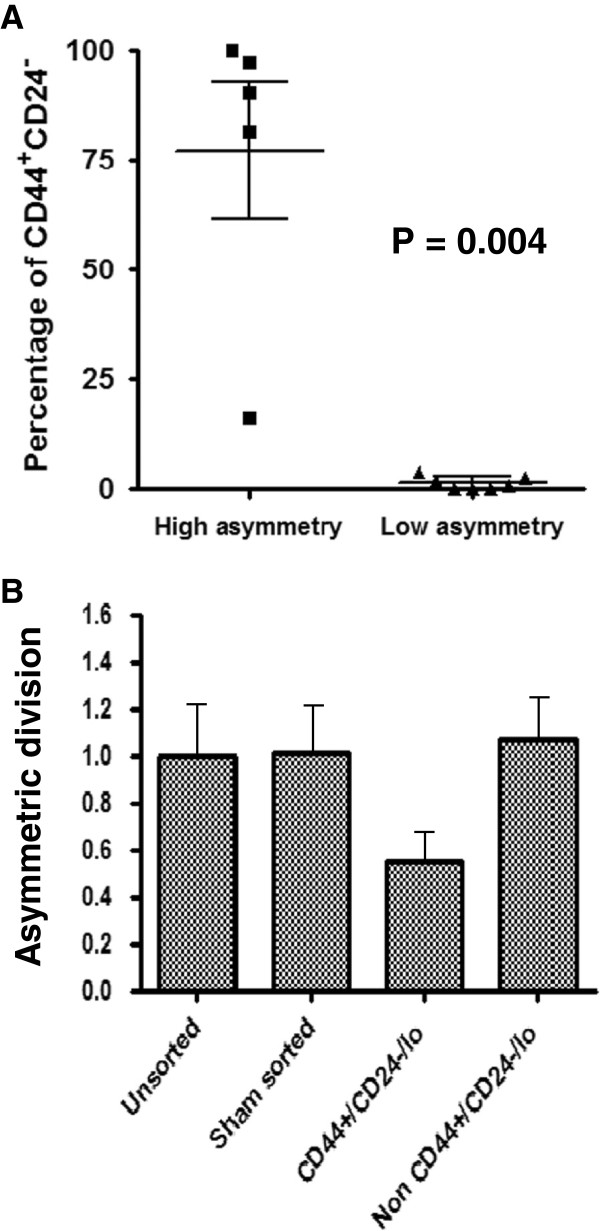
**Asymmetric segregation of template DNA is correlated with CD44**^**+**^**/CD24**^**-/lo **^**expression. (A)** Breast cancer cell lines were divided into two groups according to the percentage of BrdU asymmetric division observed, high asymmetry (mean, ≥ 0.5%) and low asymmetry (mean, < 0.5%), then plotted against the percentage of cells that were CD44^+^/CD24^-/lo^. Cell lines with high asymmetry had a higher percentage of cells that were CD44^+^/CD24^-/lo^ as compared to cell lines with low asymmetry (P = 0.004, Mann–Whitney test). Samples were analyzed in triplicates and in at least two independent experiments. **(B)** Fold-change in frequency of asymmetric segregation of template DNA in HCC1143 cells, normalized to parental-unsorted population (unsorted), are shown for cells that were sorted without gating (sham sorted), sorted stem cells (CD44^+^/CD24^-/lo^), and sorted cells negative for the CD44^+^/CD24^-/lo^ immunophenotype (not CD44^+^/CD24^-/lo^). Error bars are standard error of the mean.

It was previously hypothesized that when normal stem cells or CSCs asymmetrically segregate their temple DNA, they retain their original chromosomes and pass their newly synthesized DNA to differentiating daughter cells [13,16,17]. We therefore posited that breast cancer samples with a high percentage of CSCs would be more likely to harbor asymmetrically dividing cells. To test this, we examined breast CSC-associated surface markers by flow cytometry and performed functional CSC assays on all twelve breast cancer cell lines in our panel. We first evaluated expression of CD44 and CD24, since CD44^+^/CD24^-/lo^ is a well-established surface CSC phenotype for breast cancer as well as stem cells in normal breast tissues [27-29]. The percentage of cells within the CD44^+^/CD24^-/lo^ fraction varied substantially among different cell lines, consistent with previous reports (Table 1) [30]. Cell lines that had a consistent population of cells harboring asymmetric segregation of template DNA also had a higher percentage of CD44^+^/CD24^-/lo^ cells (P = 0.004) (Figure 3A), suggesting that cell lines with a high CD44^+^/CD24^-/lo^ fraction are enriched in cells undergoing asymmetric segregation of template DNA.

To test whether asymmetric segregation of template DNA may be enriched within the putative CSC CD44^+^/CD24^-/lo^ fraction, we examined this fraction after it was isolated by cell sorting. The sorted MCF7 CD44^+^/CD24^-/lo^ CSC fraction did not harbor any cells with detectable asymmetric segregation of template DNA (Figure 3B). To verify these findings, we sorted HCC1143 cells, whose bulk population had a larger 16.4% CD44^+^/CD24^-/lo^ population and a substantial 6.3% of cells that had asymmetric segregation of template DNA. We observed no enrichment for cells that asymmetrically segregated its template DNA within the CSC-sorted population. The CD44^+^/CD24^-/lo^ fraction had a nearly two-fold decreased frequency of asymmetric segregation of template DNA as compared to the non-CSC fraction (Figure 3B). These data suggest that the CD44^+^/CD24^-/lo^ CSC population might undergo more symmetric self-renewing divisions as compared to the bulk population within basal-like subtypes, and that asymmetric segregation of template DNA is breast cancer subtype-dependent.

High aldehyde dehydrogenase (ALDH) activity is another marker for breast CSCs and predictor of poor outcome [31]. Two isoforms, ALDH1A1 and ALDH1A3 have been implicated in driving ALDH1 activity [32,33]. We examined expression of ALDH1A1 and ALDH1A3 in our panel of 12 cell lines by flow cytometry and compared them to the frequency of asymmetric segregation of template DNA. No significant correlation was observed (ALDH1A1, P = 0.68; ALDH1A3, P = 0.12, Data not shown). Due to a lack of correlation between asymmetric segregation of template DNA and expression of the two main enzymes responsible for ALDH activity, we did not perform an ALDH activity assay on the breast cancer cell lines.

**Figure 4:**
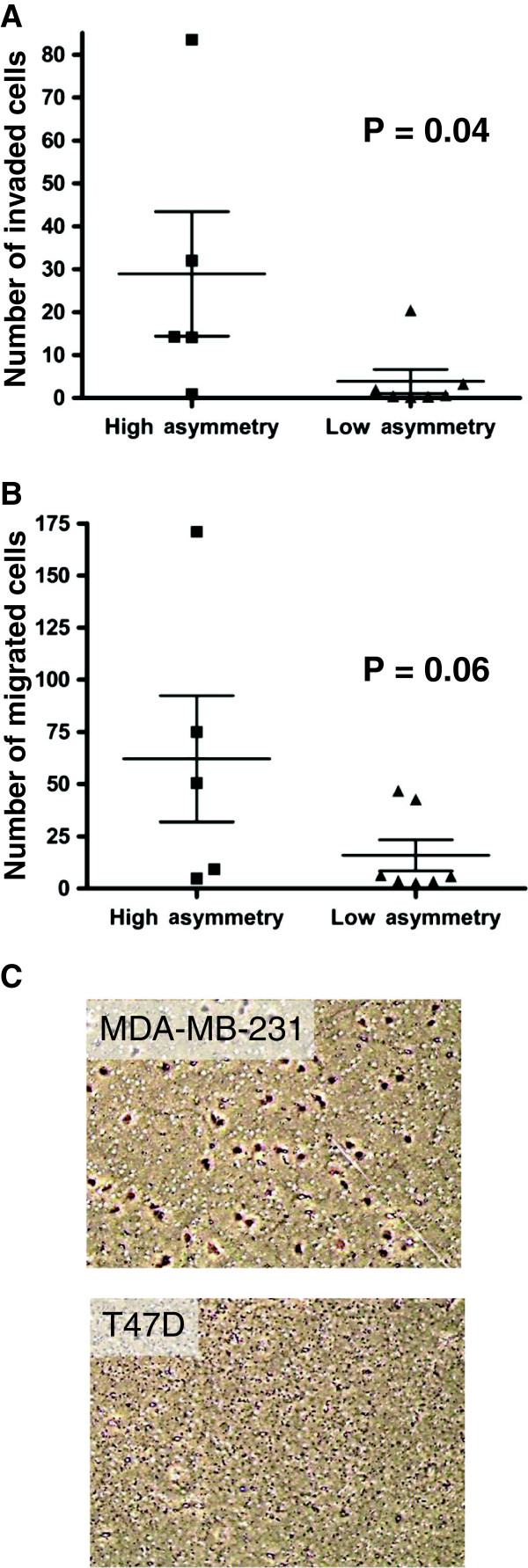
**Asymmetric segregation of template DNA is correlated with invasion and migration abilities. (A-C)** The breast cancer cell lines were subjected to invasion and migration assays, and the number of cells that invaded **(A)** and migrated **(B)** are shown. Experiments were performed in triplicates and in at least two independent assays. The breast cancer cell lines were divided into two groups according to the percentage of BrdU asymmetric division observed, high asymmetry (mean, ≥0.5%) and low asymmetry (mean, <0.5%). **(C)** Representative images of invaded MDA-MB-231 and T47D cells, fixed and stained with crystal violet, are shown.

We next evaluated invasion and migration abilities within our breast cancer panel (Figure 4A and B, Table 1). We observed a statistically significant positive correlation between the frequency of asymmetric segregation of template DNA and invasion ability (P = 0.04), and a non-significant trend with cell migration (P = 0.06), suggesting that breast cancers with the capacity for asymmetric segregation of template DNA may have an increased ability to metastasize. Soft agar colony assays were also performed to assess the clonogenic capacity of the breast cancer cell line panel. However, no correlation was observed between clonogenic capacity and asymmetric segregation of template DNA (Table 1).

### Mesenchymal stem cells promote symmetric self-renewal in breast cancer

**Figure 5:**
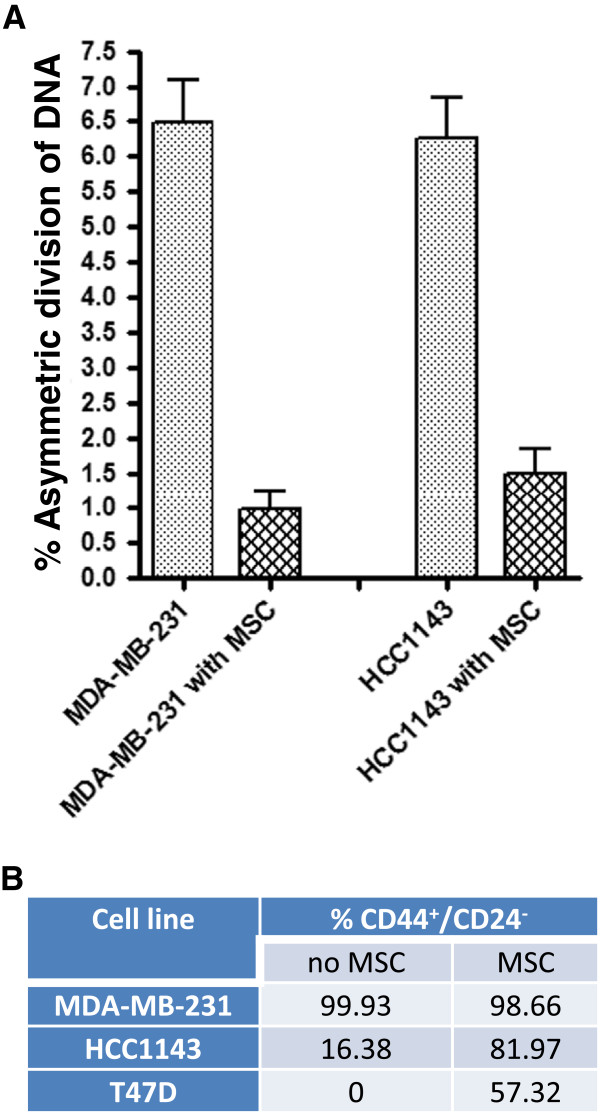
**Mesenchymal stem cells increase breast cancer cell symmetric self-renewing divisions and decreases frequency of asymmetric segregation of template DNA. (A)** Cells were grown with MSCs after BrdU was removed, and examined for changes in asymmetric segregation of template DNA. Percentages of asymmetric segregation of template DNA are shown with standard error of the mean of triplicate co-culture experiments. **(B)** The percentage of breast cancer cells expressing the CD44^+^/CD4^-/lo^ immunophenotype was quantified by flow cytometry before and after they were co-cultured with MSCs.

Mesenchymal stem cells (MSCs) are progenitors within the bone marrow stroma that have been shown to not only support breast cancer growth *in vitro* and in *vivo*[34,35], but also to expand the breast CSC population [36]. To further investigate the functional relevance of asymmetric segregation of template DNA, we quantified its frequency in breast cancer cells when co-cultured with human bone marrow-derived MSCs. For two of the cell lines that had the highest population of cells that asymmetrically segregate their template DNA, MDA-MB-231 and HCC1143, co-culture with human MSCs significantly decreased the percentage of cells that had asymmetric segregation of template DNA, from 6.47 ± 0.63% to 0.99 ± 0.28%, and from 6.25 ± 0.62% to 1.5 ± 0.35%, respectively (Figure 5A). Because co-culturing with human MSCs decreased the frequency of asymmetric segregation of template DNA, cell lines that lacked evidence for asymmetric segregation of template DNA were excluded from analysis. The decrease in frequency of asymmetric segregation of template DNA could have arisen from two scenarios: either the mother cell underwent symmetric self-renewal to give rise to two daughter cells with similar stem-like characteristics, or it underwent symmetric differentiation, to give rise to two daughter cells that are both differentiating. To test these scenarios, we co-cultured 3 breast cancer cell lines with human MSCs and analyzed expression of CD44 and CD24 (Figure 5B). We observed a substantial increase in the CD44^+^/CD24^-/lo^ cell fraction in HCC1143 and T47D cells when co-cultured with MSCs (P < 0.05). As expected, there was no increase in percentage of cells expressing CD44^+^/CD24^-/lo^ in MDA-MB-231 cells because nearly 100% of the untreated cells were CD44^+^/CD24^-/lo^. Therefore, co-culturing breast cancer cell lines with human MSCs expands the CSC population and correlates with decreased frequency of asymmetric segregation of template DNA.

## Discussion

During embryonic development and tissue homeostasis, asymmetric cell division allows stem cells to give rise to one daughter stem cell (self-renewal) and another that is destined to differentiate. It is generally believed that, in normal tissues, asymmetric self-renewal is restricted to stem or progenitor cells. We previously extended this knowledge to lung cancer [16]. Others recently observed similar findings in hepatocellular carcinoma, gastric cancer, colon cancer, and peritoneal mesothelioma [4,17,18,20]. In this study, we show evidence of asymmetric segregation of template DNA in breast cancer. This is the first report to show a marked heterogeneity in frequency of asymmetric segregation of template DNA across cell lines that correlates with molecular subtype. Consistent evidence for asymmetric segregation of template DNA was found exclusively among cell lines within the basal-like breast cancer subtype, whereas the remainder of cell lines, which were mainly luminal, showed no evidence of consistent asymmetric segregation of template DNA. More studies are needed to confirm these observations, particularly in primary breast cancer. Furthermore, studies on asymmetric cell division within different cell lineages in the mammary gland might shed light on whether the association between asymmetric segregation of template DNA and the basal-like phenotype is cell-lineage dependent or a feature arising during neoplastic development.

We observed an inverse correlation between asymmetric segregation of template DNA and cell surface Her2 expression. Since Her2 expression in breast tumors is associated with unfavorable prognosis [24], this may indicate that symmetrically dividing, self-renewing stem cells tend to be more aggressive. Furthermore, our observation is consistent with the findings by Cicalese et al., in which constitutive activation of Her2 in the ErbB2 murine breast cancer model results in excessive self-renewal and decreased asymmetric cell divisions [1]. The inverse correlation between Her2 expression and asymmetric segregation of template DNA could also be due to the fact that most Her2+ breast cancer cell lines fall into the luminal subtype [37], or that the frequency of asymmetric segregation of template DNA in Her2+ breast cancers is below our sensitivity limit of detection. In addition, breast cancer patients with an inherited TP53 mutation are more likely to present with amplification of HER2 [38], and TP53 mutations result in expansion of primary premalignant mouse mammary stem cells [1]. Since wild-type p53 increases the kinetics of immortal DNA strand co-segregation [25], the potential link between Her2 and asymmetric segregation of template DNA may be partly due to TP53 status. Additional breast cancer samples should be tested and further studies are needed to determine if Her2 expression and/or p53 actively participate in asymmetric segregation of template DNA in human breast cancer.

Cell lines with high percentages of asymmetric segregation of template DNA were enriched for cells that expressed the breast CSC-associated CD44^+^/CD24^-/lo^ markers on their cell surface, and had significantly higher invasive capability, as compared to cell lines with no or barely detectable asymmetric segregation of template DNA. These data suggest that cell lines with more CSC-like properties have higher percentages of asymmetric segregation of template DNA. This trend could also be due to the fact that basal-like cell lines harbor a higher fraction of CD44^+^/CD24^-/lo^ cells [37] and have much higher invasive ability [21]. Enriched CD44^+^/CD24^-/lo^ luminal MCF7 cells did not harbor detectable asymmetric segregation of template DNA among sorted CD44^+^/CD24^-/lo^ cells, suggesting that the differences in frequency of non-random chromosome segregation among cell lines were not due to differences in the CD44^+^/CD24^-/lo^ populations. This was supported by the fact that CD44^+^/CD24^-/lo^ cells isolated from HCC1143 cells also did not show enrichment in frequency of asymmetric segregation of template DNA. However, because the sample size was small, a larger study is warranted.

When basal-like breast cancer cells were co-cultured with bone marrow-derived MSCs, there was a marked increase in the breast CSC population [36]. When we co-cultured breast cancer cells with MSCs, we observed a marked expansion of the CD44^+^/CD24^-/lo^ CSC population, and a switch from asymmetric to symmetric segregation of template DNA. Future studies are needed to determine if MSCs increase the breast CSC pool directly by shifting the balance from asymmetric cell division to symmetric self-renewal. Furthermore, studies are needed to determine if the positive feedback cytokine loop involving IL6 and CXCL7, that has been shown to increase the breast CSC fraction within breast cancer cell lines and mouse xenografts [36], participates in the decrease in frequency of asymmetric segregation of template DNA when breast cancer cells are exposed to MSCs.

According to classical DNA analog pulse-labeling or pulse-chase assays, the DNA analog, e.g., BrdU, cannot be incorporated into the template DNA strands if the stem cell undergoes repeated non-random chromosome segregation [5,11,12,39]. However, template DNA strands are “born” in certain cases when the stem cell expands via symmetric division during normal development, injury repair, or exponential growth of cancer. We previously showed that during *in vitro* expansion of lung cancer cells, asymmetric segregation of template DNA is often interrupted by intervening symmetric divisions, and we surmised that a propensity towards excessive self-renewal via symmetric divisions among CSCs contributes to the exponential expansion of tumors [16]. Conversely, if the template DNA is not labeled during the two-week pulse period, then asymmetric segregation of the BrdU-labeled newly synthesized DNA would be observed during the first anaphase following BrdU removal. We did not observe any asymmetric BrdU segregation during the first cell division after BrdU removal in any of the cell lines (Data not shown), suggesting that both template and newly synthesized DNA were labeled during the long pulse period. Further studies would be needed to determine if the daughter cells retaining the BrdU-labeled template chromosomes are breast CSCs or differentiating daughter cells. In addition, studies involving a classical “chase” period following the BrdU pulse, in which an excess of thymidine is used to more rapidly eliminate the BrdU, may improve sensitivity.

Several hypotheses have been proposed to explain asymmetric segregation of template DNA strands. The immortal strand hypothesis was proposed decades ago by John Cairns [39] and states that normal stem cells protect the integrity of the genome by retaining the original copy of their DNA template, thus preventing the accumulation of mutations during replication. However, the proposal has been challenged by the fact that replication errors are not the only cause of DNA mutations during stem cell divisions. An alternative, though not mutually exclusive, explanation is that differences in epigenetic markers between the two copies of chromosomes direct divergent cell fates [40]. Both hypotheses support the evidence that asymmetric segregation of template DNA in stem cells is correlated with cell fate determination in daughter cells, although it is unclear if asymmetric segregation of template DNA in non-stem cells results in differing cell fates. Notwithstanding, exposure of cancer cells with an agent that increases self-renewal, i.e., MSCs, will increase the CSC pool by altering the balance between asymmetric and symmetric cell divisions. This implies that shifting the balance to symmetric differentiated divisions could deplete the CSC pool.

Although asymmetric segregation of template DNA and label-retaining cells from various organisms and tissues have been studied [5-12,41-43], there have also been reports on failure to find this phenotype [14,15,44-52]. We now show that asymmetric segregation of template DNA occurs in breast cancer cell lines, in accordance with previous lung cancer cell lines and primary lung tumors [16], as well as other cancer types [4,17,18,20]. The controversy in the literature could be partly due to differences in organisms, tissues, environment, timing as well as experimental approaches. Understanding the mechanisms driving asymmetric segregation of template DNA could clarify stem cell determination in normal tissues, and provide insights into the mechanisms of cancer cell hierarchy. Specific treatment targeting self-renewing symmetric divisions could then be a potentially effective cancer therapy.

## Materials and methods

### Cell culture

Human breast cancer cell lines MDA-MB-231, MCF7, MDA-MB-157, SKBR3 and MDA-MB-361 cells were grown in DMEM medium (Invitrogen), MDA-MB-468, T47D and MDA-MB-453 were grown in DMEM/F12 medium (Invitrogen) and BT549, HBL100, HCC1954 and HCC1143 were grown in RPMI medium (Invitrogen). All growth media were supplemented with 100U/ml penicillin, 0.1 mg/ml streptomycin (Invitrogen), 2 mM glutamine (Invitrogen), and 10% FBS (Sigma). Cells were cultured in a standard incubator under ambient O_2_ concentrations with 5% CO2 at 37°C. All cell lines tested were authenticated by Genetica DNA Laboratories.

### Asymmetric segregation of template DNA assay

Asymmetric segregation of template DNA was quantified exactly as described [16]. Briefly, cells were cultured in the presence of 1 μM BrdU for at least 2 weeks to ensure all the cells were labeled. Cells were then cultured for two population doublings in the absence of BrdU, and then were either collected by mitotic shake-off, or stained directly on glass slides. Population doubling times were determined beforehand in at least two independent experiments, under the same conditions as used for detecting asymmetric segregation of template DNA. Mitotic cells were fixed in cold 70% ethanol for at least 30 min., then exposed to 2 N HCl with 0.5% Triton-X-100 for 1 hr. After washing, cells were incubated with a 1:10 dilution of anti-BrdU-FITC (BD Biosciences). Slides were washed, dried and mounted using Vectashield containing DAPI (Vector Laboratories) [16].

BrdU asymmetric division was imaged and manually scored by visualization of anaphase cells using a Nikon TE200 microscope with DS-Ri1 camera and NIS-Elements BR software. Cells were scored as described in detail [16]. Complete BrdU segregation to one set of chromosomes and absence of any BrdU in the other was considered as asymmetric segregation of template DNA. For every cell line, scoring was performed on three replicates, each with ≥200 anaphase cells, and repeated at least once.

### Flow cytometry and cell sorting

Cells were trypsinized, washed with PBS/0.5% BSA, then primary antibody was added and incubated for 30 min on ice. After washing, 7-AAD (BD Biosciences) was added as a viability dye. IgG control-stained cells were used for setting gates. For detection of ALDH1A1 and ALDH1A3, cells were fixed then permeabilized in 4% paraformaldehyde followed by 0.5% Triton-X-100 before incubation with the primary antibody. Secondary antibody was Alexafluor 488 anti-Rb (Invitrogen). Analysis was performed on a FACscalibur flow cytometry machine using CellQuest Pro software. Antibodies used were: APC-conjugated anti-CD44 (BD Pharmigen); FITC-conjugated anti-CD24 (BD Pharmingen); APC-conjugated anti-ErbB2 (R&D Systems); rabbit anti-ALDHA1 (ABGENT); rabbit anti-ALDHA3 (ABGENT).

For cell sorting experiments, cells were cultured in the presence of 1 μM BrdU for at least 2 weeks and then stained as above for flow cytometry, then sorted using a BD Biosciences Influx High Speed Cell Sorter. Post-sort purity checks confirmed the purity of the sorted populations. Sorted cells were cultured in the absence of BrdU for 2 population doublings, and then examined for asymmetric segregation of template DNA, as explained above.

### Invasion and migration assay

Eight micron cell culture inserts were placed in companion plates (BD Biosciences). For invasion assays, cell culture inserts were coated with Matrigel (BD Biosciences) diluted to 1g/L in serum-free cell culture media for the specific cell line. For both invasion and migration assays, cells were harvested using trypsin and washed twice in cell culture media without serum. Cells, 1 × 10^5^ or 1.25 × 10^4^, were added to the invasion or migration chambers, respectively. The lower chambers were filled with 0.6 ml of cell culture media with 10% FBS as a chemo-attractant. Chambers were then incubated at 37°C for 24 hrs. Cells on the upper surface of the inserts were removed gently using cotton swabs. Cells on the lower surface were fixed and stained in 0.05% crystal violet for 10 min. Counting of the invaded or migrated cells was performed using a 10X objective on a Nikon TMS microscope on five independent fields. All experiments were done in triplicate and repeated at least once. If there were too few cells in each field of view during counting, the starting number of plated cells were then doubled or quadrupled.

### Soft agar colony assay

Cells (1 × 10^4^) were suspended in 0.425% Noble agar (BD Biosciences) in culture medium above a 0.625% agar-medium base layer in 6 cm dishes. An additional 0.625% agar-medium layer was applied above the cell layer. Agar plates were incubated in a standard cell culture incubator, were topped with 1 ml complete cell culture media that was replaced once a week for 3 weeks, and then stained in 0.005% crystal violet for 1 hr. Plates were scanned using MP Navigator EX1.0 software using a Canon 8800F scanner. Colonies with 10 or more cells in diameter were counted using ImageJ software ( http://rsbweb.nih.gov/ij). Every cell line was assayed in two independent experiments, each with three replicates.

### Co-culture of breast cancer cells with MSCs

Human bone marrow-derived MSCs harvested from pooled normal human donors were purchased from Lonza, and expanded for 3 passages in Mesenchymal Stem Cell Growth Medium (Lonza). For co-culture flow-cytometry experiments, MSCs were labeled with Cell Tracker Red (CTR) (Invitrogen) and grown with breast cancer cells for 3 days in the cell culture media used for the cell line, such that the final confluence of the flask was ≤ 90%. Flow cytometry on co-cultured breast cancer cells was performed as described above, except the MSCs were excluded from analyses by gating out CTR fluorescence. Asymmetric segregation of template DNA during cell division was quantified by a colony-forming assay as described. Specifically, 300 breast cancer cells that were grown in the presence of 1 μM BrdU for at least two weeks were labeled with CTR and then plated at clonal density on glass slides with a final 1:1 ratio of the same cell line (not BrdU-pulsed or CTR-labeled) and MSCs, such that the confluence at the end of the assay was ≤ 90%. Slides were fixed and stained once small colonies (4–6 cells) were formed. Asymmetric segregation of template DNA was determined based on inheritance pattern of BrdU in the progeny, as described [16].

### Statistical analysis

Statistics were performed using Stata software, version 12. Comparison between asymmetric segregation of template DNA and breast cancer molecular subtype was done by a Fisher’s exact test. Comparison between asymmetric cell division and Her2 MFI, and percentage of cells with CD44^+^/CD24^-/lo^ expression, invasion and migration capacity, and colony formation was performed by a non-parametric Mann–Whitney test. P values 0.05 or below were considered statistically significant.

## Competing interests

The authors declare that they have no competing interests.

## Authors’ contributions

BMR and SRP designed the experiments. WL, GJ, SA, KM and SRP performed the experiments. WL and SRP analyzed the data, interpreted the results, and wrote the manuscript. All authors read and approved the final manuscript.
